# Panorama phylogenetic diversity and distribution of type A influenza viruses based on their six internal gene sequences

**DOI:** 10.1186/1743-422X-6-137

**Published:** 2009-09-08

**Authors:** Ji-Ming Chen, Ying-Xue Sun, Ji-Wang Chen, Shuo Liu, Jian-Min Yu, Chao-Jian Shen, Xiang-Dong Sun, Dong Peng

**Affiliations:** 1 The Laboratory of Animal Epidemiological Surveillance, China Animal Health & Epidemiology Center, Qingdao, PR China; 2 The Feinberg School of Medicine, Northwestern University, Chicago, USA

## Abstract

**Background:**

Type A influenza viruses are important pathogens of humans, birds, pigs, horses and some marine mammals. The viruses have evolved into multiple complicated subtypes, lineages and sublineages. Recently, the phylogenetic diversity of type A influenza viruses from a whole view has been described based on the viral external HA and NA gene sequences, but remains unclear in terms of their six internal genes (PB2, PB1, PA, NP, MP and NS).

**Methods:**

In this report, 2798 representative sequences of the six viral internal genes were selected from GenBank using the web servers in NCBI Influenza Virus Resource. Then, the phylogenetic relationships among the representative sequences were calculated using the software tools MEGA 4.1 and RAxML 7.0.4. Lineages and sublineages were classified mainly according to topology of the phylogenetic trees and distribution of the viruses in hosts, regions and time.

**Results:**

The panorama phylogenetic trees of the six internal genes of type A influenza viruses were constructed. Lineages and sublineages within the type based on the six internal genes were classified and designated by a tentative universal numerical nomenclature system. The diversity of influenza viruses circulating in different regions, periods, and hosts based on the panorama trees was analyzed.

**Conclusion:**

This study presents the first whole views to the phylogenetic diversity and distribution of type A influenza viruses based on their six internal genes. It also proposes a tentative universal nomenclature system for the viral lineages and sublineages. These can be a candidate framework to generalize the history and explore the future of the viruses, and will facilitate future scientific communications on the phylogenetic diversity and evolution of the viruses. In addition, it provides a novel phylogenetic view (i.e. the whole view) to recognize the viruses including the origin of the pandemic A(H1N1) influenza viruses.

## Background

Type A influenza viruses can infect humans and many kinds of animals including birds, pigs, horses and some marine animals [1]. The viruses host eight segments in its genome. The fourth and sixth segments encode the viral external genes, HA and NA, respectively. The other six segments encode the viral internal genes, PB2, PB1, PA, NP, MP and NS, respectively. The PB1, MP and NS genes each encode two overlapping proteins (i.e. PB1-F2 overlapping with PB1, M2 overlapping with M1, NS2 overlapping with NS1). According to the viral external HA and NA gene sequences and their serological features, type A influenza viruses have been classified into 16 HA subtypes (H1-H16) and 9 NA subtypes (N1-N9) [2,3]. The combinations of the HA and NA subtypes further formed dozens of subtypes including H1N1, H1N2, H2N2, H3N2 and H5N1. In addition, according to each of the viral internal gene sequences, the viruses have been classified into some lineages and sublineages, such as the North American lineage, the gull lineage, the human-like swine lineage, etc [4-9].

In the past century, type A influenza viruses have become highly diversified and complicated mainly through natural point mutations, cross-host transmission and genomic segment re-assortment among or within the subtypes, lineages or sublineages [1-47]. Consequently, sometimes it is difficult to locate a new influenza virus in the viral family and trace its origin.

Recently, the panorama phylogenetic trees of type A influenza viruses based on their external HA and NA gene sequences were described, which could be used as the "maps" in tracing an influenza virus through phylogenetic analysis of the two genes [3]. However, their phylogenetic diversity from a whole view largely remains unclear in terms of their six internal genes, though many papers have been published on their phylogenetic diversity of limited time, regions or hosts [4-9,15-39].

The six internal genes of type A influenza viruses were important in phylogenetic analysis, as demonstrated below in tracing the origin of the pandemic influenza virus recently emerging in Mexico [41,42]. The new virus was designated as A(H1N1) influenza virus by World Health Organization and has spread to many countries. Some experts claimed that the virus was an unusually mongrelized mix of human, avian and swine influenza viruses with the PB2 and PA genes from avian viruses and the PB1 gene from human viruses, while some others assumed that all the genes were from swine influenza viruses. The latest reports indicated that both opinions were somehow rational [40-47]. Here, we report the panorama phylogenetic trees of type A influenza viruses based on their six internal genes, in order to further clarify the origin of the A(H1N1) influenza virus from a new dimension, and establish a candidate framework for future scientific communications on the phylogenetic diversity and evolution of the viruses.

## Results

### Statistics of sequences type A influenza viruses

Up to May, 20, 2009, 98261 sequences of type A influenza viruses were available in GenBank. More than half of them (61528) were from USA (36887), China (mainland: 12592, Hong Kong SAR: 5656), Australia (3444) and Canada (2949). Additionally, most of them (96248) were from humans (47958), birds (42282), pigs (4846) and horses (1162), and most of them (86254) were from the viruses isolated in or after the year 1990.

Up to May, 20, 2009, 7189 PB2, 7226 PB1, 7074 PA and 7238 NP sequences (≥ 300 amino acid residues) as well as 7954 NS1 and 8605 MP sequences (≥ 150 amino acid residues) were available in GenBank. They were taken as the candidates of the representative sequences.

### The panorama phylogenetic trees of the six internal genes

**Figure 1:**
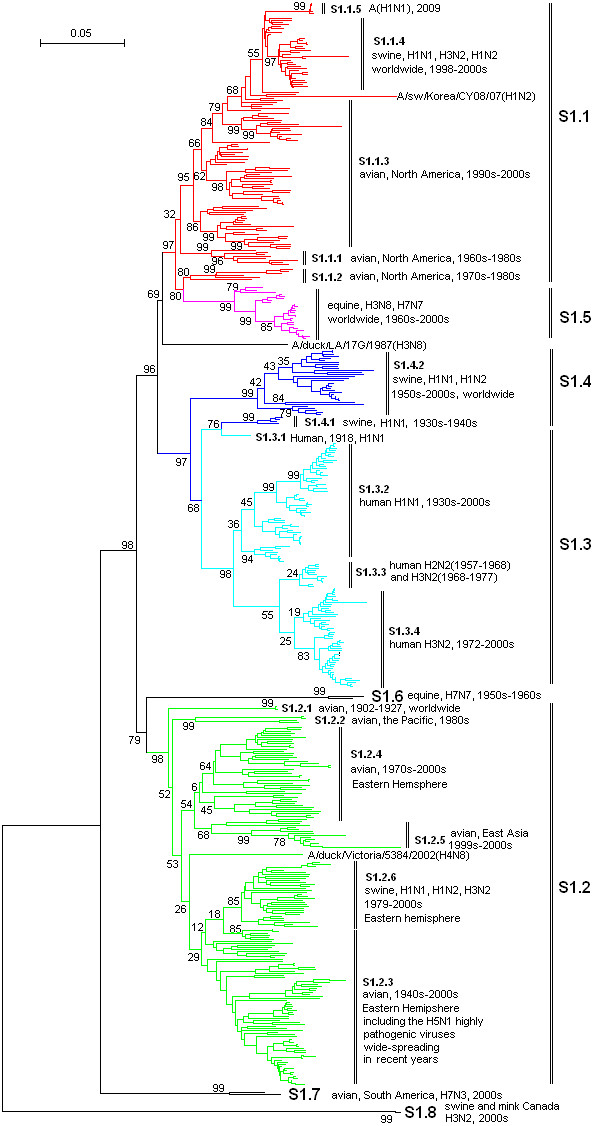
**The panorama phylogenetic tree of type A influenza virus based on the viral PB2 gene sequences**. The tree could be divided into at least 8 lineages, and some lineage could be further divided into some sublineages. The distribution of host, isolation time, isolation regions and subtypes of the majority within each sublineages were shown near to the relevant designations. The current A(H1N1) virus corresponded to the sublineage S1.1.5 (at the top). Bootstrap values were given at relevant nodes.

**Figure 2:**
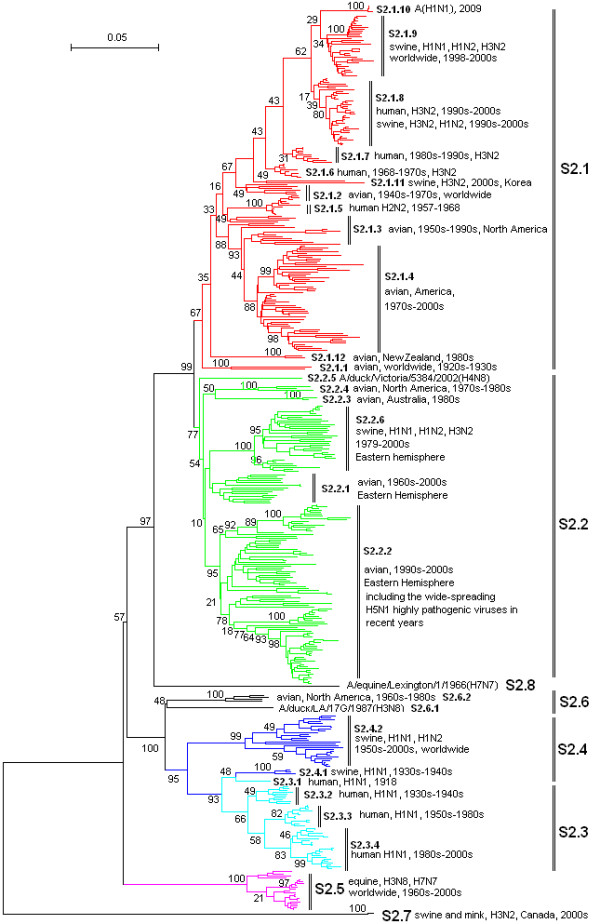
**The panorama phylogenetic tree of type A influenza virus based on the viral PB1 gene sequences**. The tree could be divided into at least 8 lineages, and some lineage could be further divided into some sublineages. The distribution of host, isolation time, isolation regions and subtypes of the majority within each sublineages were shown near to the relevant designations. The current A(H1N1) viruses were within the sublineage S2.1.10 (at the top). Bootstrap values were given at relevant nodes.

**Figure 3:**
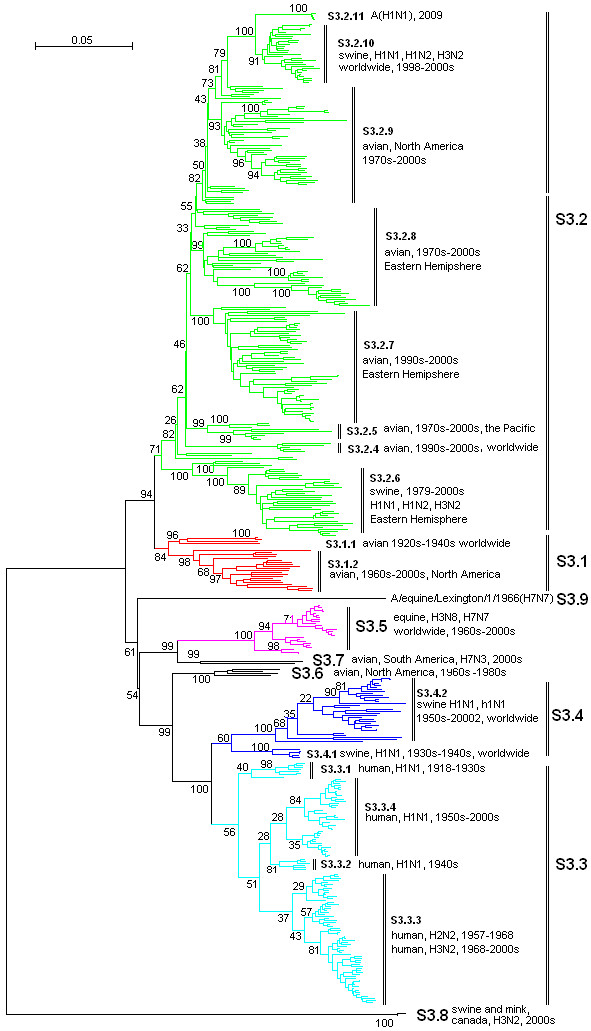
**The panorama phylogenetic tree of type A influenza virus based on the viral PA gene sequences**. The tree could be divided into at least 9 lineages, and some lineage could be further divided into some sublineages. The distribution of host, isolation time, isolation regions and subtypes of the majority within each sublineages were shown near to the relevant designations. The current A(H1N1) virus corresponded to the sublineage S3.2.11 (at the top). Bootstrap values were given at relevant nodes.

**Figure 4:**
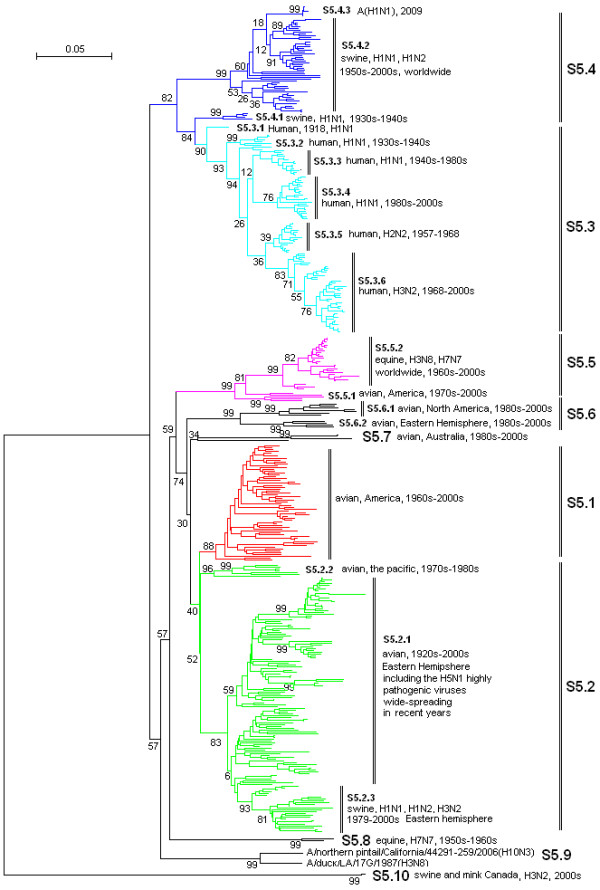
**The panorama phylogenetic tree of type A influenza virus based on the viral NP gene sequences**. The tree could be divided into at least 10 lineages, and some lineage could be further divided into some sublineages. The distribution of host, isolation time, isolation regions and subtypes of the majority within each sublineages were shown near to the relevant designations. The current A(H1N1) virus corresponded to the sublineage S5.4.3 (at the top). Bootstrap values were given at relevant nodes.

**Figure 5:**
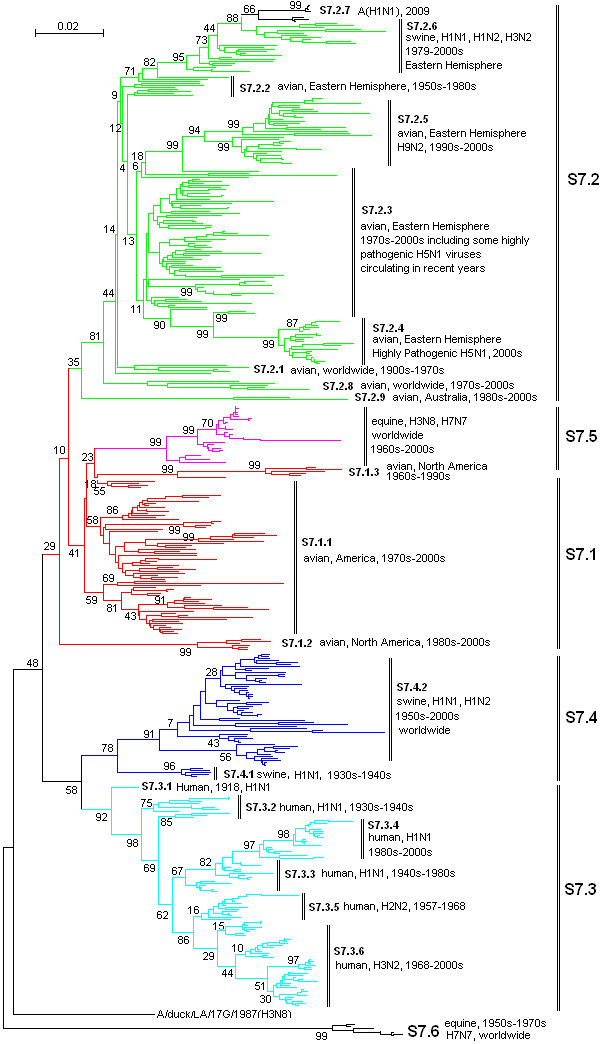
**The panorama phylogenetic tree of type A influenza virus based on the viral MP gene sequences**. The tree could be divided into at least 6 lineages, and some lineage could be further divided into some sublineages. The distribution of host, isolation time, isolation regions and subtypes of the majority within each sublineages were shown near to the relevant designations. The current A(H1N1) virus corresponded to the sublineage S7.2.7 (at the top). Bootstrap values were given at relevant nodes.

**Figure 6:**
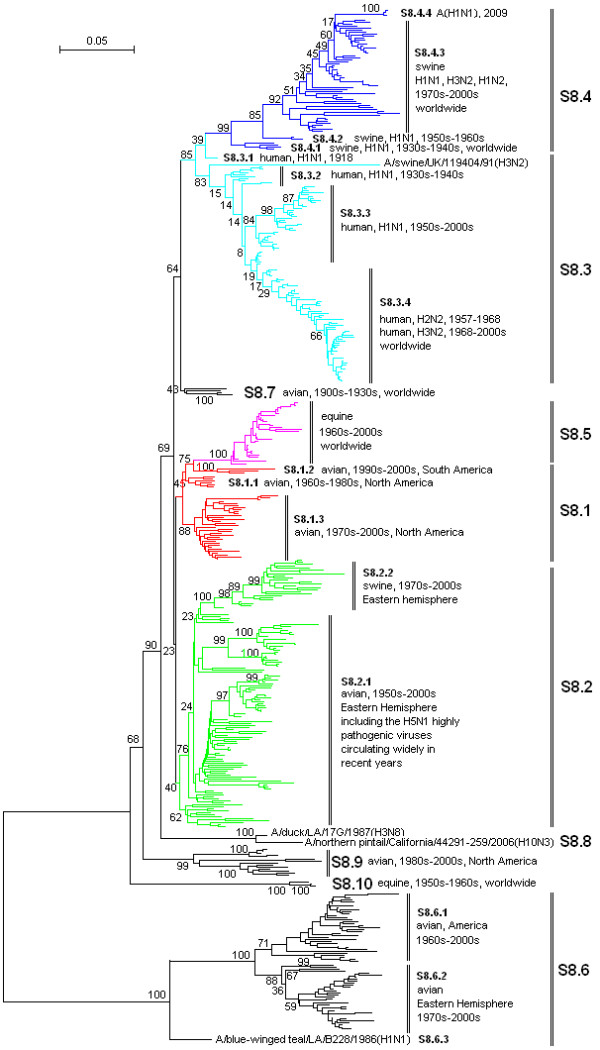
**The panorama phylogenetic tree of type A influenza virus based on the viral NS gene sequences**. The tree could be divided into at least 10 lineages, and some lineage could be further divided into some sublineages. The distribution of host, isolation time, isolation regions and subtypes of the majority within each sublineages were shown near to the relevant designations. The current A(H1N1) virus corresponded to the sublineage S8.4.4 (at the top). Bootstrap values were given at relevant nodes.

2798 (492 PB2, 450 PB1, 471 PA, 436 NP, 473 M, 476 NS) representative sequences were selected. Their designations and alignment were given in additional files 1, 2, 3, 4, 5 and 6, respectively. Over half of them were from the same viruses. Their phylogenetic trees were shown by Figures 1, 2, 3, 4, 5 and 6, respectively. The original tree files with virus designations were given in additional files 7, 8, 9, 10, 11, and 12, respectively.

Figures 1, 2, 3, 4, 5 and 6 showed that the sequences of each of the viral genes could be divided into 6-10 lineages, and some of the lineages could be further divided into several sublineages. The distribution of the lineages and sublineages in hosts, isolation time and places were given in the figures without description of the exceptions (most of the exceptions were marked with asterisks in additional files 7, 8, 9, 10, 11, and 12). They were all located in separated branches in the phylogenetic trees and most of them were of high bootstrap values (>70). Some lineages or sublineages like S2.1 and S2.2 were of low bootstrap values presumably due to the existence of intermediate sequences [2,48].

### The similarity of the phylogenetic trees of the six internal genes

Figures 1, 2, 3, 4, 5 and 6 suggested that, with more or fewer exceptions, the first lineage of the six internal genes (S1.1, S2.1, S3.1, S4.1, S5.1 and S6.1) all largely corresponded to avian influenza viruses isolated from the Western Hemisphere (North and South America). The second lineage of the six internal genes (S1.2, S2.2, S3.2, S4.2, S5.2 and S6.2) all largely corresponded to avian influenza viruses isolated from the Eastern Hemisphere (Europe, Asia, Africa and the Pacific). The third lineage of the six internal genes (S1.3, S2.3, S3.3, S4.3, S5.3 and S6.3,) all largely corresponded to seasonal human influenza viruses. The fourth lineage (S1.4, S2.4, S3.4, S4.4, S5.4 and S6.4) all largely corresponded to classical swine influenza viruses. The fifth lineage (S1.5, S2.5, S3.5, S4.5, S5.5 and S6.5,) all largely corresponded to equine H3N8 or H7N7 influenza viruses isolated in the 1960s-2000s. These five lineages covered most of the representatives for each of the six internal genes.

The distribution of the main lineages of human (S1.3, S2.3, S3.3, S4.3, S5.3 and S6.3), swine (S1.4, S2.4, S3.4, S4.4, S5.4 and S6.4) and equine swine (S1.5, S2.5, S3.5, S4.5, S5.5 and S6.5) influenza viruses in isolation places and isolation time were consistent among the six internal genes except that, as for the PB1 gene, subtypes H2N2 and H3N2 human influenza viruses were located in the avian lineage S2.1 rather than in the human lineage S2.3.

### The heterogeneity of the phylogenetic trees of the six internal genes

The phylogenetic trees were also somehow different among the six internal genes, especially for avian influenza viruses. The most striking heterogeneity was that avian influenza viruses were largely divided into two lineages corresponding to the two hemispheres, respectively, based on the viral PB2, PB1, PA, NP and MP gene sequences (Figures 1, 2, 3, 4 and 5), but based on the viral NS gene, they could be divided into two clusters each of which could be further divided into two lineages or sublineages corresponding to the two hemispheres, respectively (Figure 6). This is consistent with a previous report [8]. Another striking heterogeneity was that, based on the viral PA gene, the avian lineage S3.1 corresponding to the Western Hemisphere was too small and many viruses isolated in America in the 1970s-2000s were located in the lineage S3.2 which was mainly corresponding to the viruses isolated in the Eastern Hemisphere in the 1920s-2000s (Figure 3). In addition, the lineage S1.7 covered a few H7N3 subtype avian influenza viruses isolated from South America based on the viral PB2 gene (Figure 1). However, they were only a small branch (marked with black triangles in additional file 8) within the sublineage S2.1.4 along with some avian influenza viruses isolated in the 1990s in North America based on the viral PB1 gene.

### The diversity of influenza viruses circulating in different regions based on the six internal genes

Like the panorama phylogenetic trees based on the viral HA and NA genes [3], the ones reported here based on the six internal genes (Figures 1, 2, 3, 4, 5 and 6) suggested that human and equine influenza viruses differed little among regions, but avian influenza viruses demonstrated obvious geographical differences. Many avian influenza viruses isolated in the same hemisphere were situated in the same lineages or sublineages, and many avian influenza viruses isolated in different hemispheres were situated in different lineages or sublineages.

### The diversity of influenza viruses circulating in different time based on the six internal genes

Figures 1, 2, 3, 4, 5 and 6 suggested that, based on the six internal gene sequences, all the influenza viruses isolated from human, horses, pigs or birds showed more or less time difference, e.g. the human H3N2 influenza viruses isolated in the 1970s were different from those isolated in the 2000s. The time difference among human and equine influenza viruses was more obvious than swine influenza viruses. Avian influenza viruses showed less time difference, i.e. some avian influenza viruses were similar to each other, even though they were isolated in different time periods (like A/turkey/England/N28/73(H5N2) and A/chicken/Hebei/1/2002(H7N2) in terms of the PB2 gene in additional file 7), and some avian influenza viruses within the same lineage or sublineage were quite different from each other even though they were isolated in the same period and place (e.g. A/quail/Hong Kong/G1/97(H9N2) and A/goose/Hong Kong/w222/97(H6N7) in terms of the PB2 gene in additional file 7).

### The diversity of influenza viruses circulating in different hosts based on the six internal genes

Figures 1, 2, 3, 4, 5 and 6 provided us a whole view on the diversity of equine, human and swine influenza viruses. As consistent with the viral HA and NA genes [1,3], the diversity of influenza viruses isolated from horses was simple without much divergence, and H7N7 subtype equine influenza viruses disappeared from the earth at the end of the 1970s [1]. Human influenza viruses were more complicated than equine influenza viruses in diversity. They were divided into H1N1, H2N2, H3N2 subtypes each of which, however, diverged into few co-existing sublineages [26]. Avian influenza viruses were of higher diversity than human influenza viruses. They diverged into multiple lineages and sublineages, and most of them contained many viruses distinct from each other in terms of genetic distances.

Swine influenza viruses were also of high phylogenetic diversity. They could be divided into at least three major genotypes each of which were of multiple subtypes, as described below. In addition, pig infections with avian, human and equine influenza viruses were not rare, and a few swine influenza viruses such as A/swine/Quebec/4001/2005(H3N2) were strange in their gene sequences (additional file 8, 9, 10, 11 and 12).

### Three genotypes of swine influenza viruses based on the viral six internal genes

The phylogenetic trees based on the viral HA and NA genes reported previously [3], and the ones based on the six internal genes reported here, each have classified swine influenza viruses into several lineages and sublineages (Figures 1, 2, 3, 4, 5 and 6, additional files 13 and 14). The combination of the six internal genes presented us three major genotypes of swine influenza viruses circulating in the world in the past decades.

The first genotype is the classical swine influenza viruses circulating worldwide at least from the 1930s-2000s. This genotype is equal to the whole or a part of the lineage S1.4, S2.4, S3.4, h1.3, S5.4, n1.3, S7.4, S8.4 of the relevant genes (Figures 1, 2, 3, 4, 5 and 6, additional files 13 and 14), respectively, with representatives A/swine/Iowa/15/1930(H1N1) and A/swine/Iowa/15/1985(H1N1).

The second genotype is the avian-like or so-called "Eurasian" lineage swine influenza viruses presumably emerging in Europe in the 1970s and circulating only in Eurasia till date with representatives A/swine/Belgium/WVL1/1979(H1N1) and A/swine/England/WVL16/1998(H1N1). This genotype is equal to the sublineage S1.2.6, S2.2.6, S3.2.6, h1.1.3, S5.2.3, n1.1.7, S7.2.6, S8.2.2 of the relevant genes (Figures 1, 2, 3, 4, 5 and 6, additional files 13 and 14), respectively. Its eight genomic segments all came from avian influenza viruses circulating in the Eastern Hemisphere.

The third genotype is the re-assortant swine influenza viruses presumably emerging in the 1990s and circulating worldwide [49]. It corresponds to the whole or a part of the lineages S1.1.4, S2.1.9, S3.2.10, h1.3.2, S5.4.2, n1.3.2, S7.4.2, S8.4.3 of the relevant genes (Figures 1, 2, 3, 4, 5 and 6, additional files 13 and 14), respectively. The NP, NS and MP genes of the genotype were from the first genotype swine influenza viruses. The PB1 gene of the genotype was from human H3N2 viruses and the PB2 and PA genes of the genotype were both from avian influenza viruses. Viruses within this genotype include A/swine/Korea/CAS08/2005(H1N1), A/swine/Korea/JL01/2005(H1N2), A/swine/Korea/CAN04/2005(H3N2), A/swine/Minnesota/sg-00240/2007(H1N1), A/swine/Minnesota/sg-00239/2007(H1N2), A/swine/Minnesota/sg-00237/2007(H3N2).

The majority of the viruses from the first genotype were H1N1 and H1N2 subtypes of swine viruses. The majority of the viruses from the second and third genotypes were H3N2, H1N2 and H1N1 subtypes of swine viruses. A few H3N1 subtype isolates were also identified in the third genotypes. In addition, as showed by the aforementioned isolates in the third genotype, multiple subtypes of swine influenza viruses within the same genotype could circulate in the same region in the same year.

### The origin of the new A(H1N1) influenza virus emerging in North America in 2009

**Figure 7:**
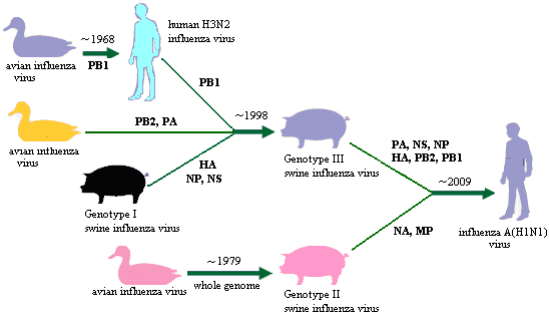
**The putative evolutionary history of the eight genes of the new A(H1N1) virus**.

The sublineage of the new A(H1N1) influenza virus was situated at the top of the six panorama phylogenetic trees (Figures 1, 2, 3, 4, 5 and 6). From the panorama phylogenetic trees and additional files 13 and 14, as given in Figure 7, the NA and M gene of the new virus should be from the aforementioned second genotype of swine influenza viruses circulating in Eurasia from 1979 to the 2000s. The other six internal genes (PB2, PB1, PA, HA, NP and NS) of the new virus should be from the third genotype of swine influenza viruses circulating worldwide from 1998 to the 2000s which hosted genes from human, avian and swine influenza viruses. In addition, five genes (PB2, PB1, PA, NA and MP) of the new virus could be traced back to avian influenza viruses, and the evolution of the PB1 gene had an additional stop in human populations.

### Cross-species transmission in the evolution of type A influenza viruses

Additional files 7, 8, 9, 10, 11 and 12 suggested that horses were seldom infected with influenza viruses of other hosts, and birds were seldom infected with mammalian influenza viruses. However, it was not rare for pigs to be infected with avian or human influenza viruses and humans to be infected with swine influenza viruses. However, human infections with an avian influenza virus were still rare except for the H5N1 highly pathogenic avian influenza viruses circulating in the Eastern Hemisphere in recent years.

### The phylogenetic trees calculated using the maximum likelihood model

The phylogenetic trees calculated using the maximum likelihood model were of no obvious difference from those calculated using the neighbor-joining model, regarding to the clades of bootstrap values higher than 70, and the lineages and sublineages classified herein were also rational for the trees calculated using the maximum likelihood model. Additional file 15 is an example, which shows the panorama phylogenetic tree of the viral PB2 gene calculated using the maximum likelihood model.

## Discussion

### Calculation and readout of the phylogenetic trees

This report plus a previous one [3] constitute the whole phylogenetic views of all the segments of the viral genomes. The web servers of Influenza Virus Resource in NCBI simplified greatly in the calculation of the phylogenetic trees. Otherwise, it should take several years to finish the work. The trees could not be calculated or correctly calculated if the sequences were shorter than a threshold. Therefore, all the representative sequences were of certain length limitation.

The representative sequences were not selected according to the size and composition of the lineages or sublineages, and thus the trees could not give the actual size and composition of the lineages and sublineages. In fact, the sequences which are specially distributed in hosts, regions and time were of higher probabilities to be selected as the representatives than common ones.

### Explanations of the heterogeneities of the panorama phylogenetic trees

The heterogeneities among the panorama phylogenetic trees of the six internal genes could be partially explained as the results of re-assortment of the viral genes [1,11]. For example, subtypes H2N2 and H3N2 human influenza viruses obtained their PB1 gene from an avian influenza virus through re-assortment [22]. Therefore, they were located within the avian lineage rather than a human lineage based on the viral PB1 gene. In principle, re-assortment of influenza viruses could occur between or within subtypes, lineages and sublineages. Re-assortment might be a partial basis of the second striking heterogeneity observed in avian influenza viruses mentioned above. That is to say, it is possible that the PA gene of an avian influenza virus from the Eastern Hemisphere flowed into the Western Hemisphere in the 1970s, and replaced the PA gene of many avian influenza viruses circulating in the Western Hemisphere through many times of re-assortment, and became established there in multiple subtypes thereby. This assumption was supported by a newly published paper [28], and additional file 9 which showed that some avian influenza viruses isolated in the Eastern Hemisphere in the 1970s within the sublineage S3.2.8 were quite similar to those isolated in the Western Hemisphere in the same period within the sublineage S3.2.9.

Heterogeneity of the lineages in mutation rates and divergence also could cause the differences in the structures of the phylogenetic trees. This might be a partial basis of the first striking heterogeneity observed in avian influenza viruses mentioned above.

### Cross-species transmission and the role of pigs in the evolution of type A influenza viruses

In principal, the frequencies of the cross-host infections are positively correlated to multiple parameters including the population size of the donor hosts and the receiver hosts as well as their contact opportunities. In addition, the cross-host infections are highly restricted if the cell-receptor of the receiver hosts do not match the viruses from the donor hosts [50,51]. Pigs are of large populations in many regions in the world and have many opportunities to contact birds and humans. Their cell-receptors match both human and avian influenza viruses [50]. Therefore, it is rational that pig infections with avian or human influenza viruses are frequently identified. For this reason, pigs should play an important role in the emergence of new human influenza viruses as an "incubator" of them. On the other hand, horses are of smaller populations and of cell-receptors matching avian rather than swine or human influenza viruses [51]. Therefore, it is reasonable to identify here that horses were seldom infected with influenza viruses of other hosts (additional files 7, 8, 9, 10, 11 and 12), and the equine lineages or sublineages are genetically closer to the avian ones than others in Figures 1, 2, 3, 4, 5 and 6.

### The significance of the panorama phylogenetic trees of the six internal genes

The panorama phylogenetic trees of the six internal genes reported here provided us not only a whole view on the diversity of type A influenza viruses, but also a candidate framework to generalize the history and explore the future of the viruses. The human pandemics of H2N2 subtype in 1957 and H3N2 subtype in 1968 [19,22], the severe outbreak of equine influenza with an avian-like H3N8 virus in China in 1989 [27], and the emergence of the new A(H1N1) virus in 2009 [41-47], all could be marked in the panorama "maps". As for the future, it is easier with the guide of the panorama "maps" to determine whether an isolate is special or odd in epidemiology (like A/duck/Victoria/5384/2002(H4N8) in lineage S1.2 in Figure 1) [52], and whether an isolate is originated from genetic re-assortment. As showed by the example in tracing the origin of the new A(H1N1) influenza virus, the panorama phylogenetic trees indicated that all the genes in the pandemic virus were directly from swine influenza viruses, but if based on some partial phylogenetic trees, we might wrongly conclude that the viral PB1 and PB2 genes were directly from a human virus and an avian influenza virus, respectively.

The panorama phylogenetic trees also gave us some novel information to recognize type A influenza viruses. Firstly, avian influenza viruses of each subtype were usually classified into Eurasian and North American lineages in the past presumably due to confinement of birds to the distinct flyways of each hemisphere [1,20,28], and here the two lineages were confirmed by the sequences of the viral PB2, PB1, PA, NP, and M genes, but their geographical distribution should be enlarged to the Eastern and Western Hemisphere, respectively. Moreover, the avian influenza viruses based on the viral NS genes were more complicated than the two lineages as mentioned above [8]. Secondly, most of the sequences of the six internal genes are of somehow host, geographical, time features. This could be utilized in the identification of an influenza virus, i.e. sequencing an internal gene of an influenza virus sometimes could indicate the profile of the virus. For example, if the sequence of the NS gene of an influenza virus belongs to S1.5, it is likely for the virus to be an equine influenza virus. Since the six internal genes are more conserved than the external HA and NA genes, identification of an influenza virus based on the internal genes could be easier than on the external genes. Thirdly, the panorama trees offered us a new dimension to recognize that pigs may play an important role in the ecology and epidemiology of type A influenza viruses of different hosts. Fourthly, the representative sequences given in additional files 1, 2, 3, 4, 5 and 6 for constructing the panorama trees could be easily visualized using the software MEGA, and the variable or the conserved regions in the genes could be easily found using the "Color Cells" function in the menu of "Display" of the software tool. Such information is useful at least for PCR primer design.

### The bias of the panorama phylogenetic trees of the six internal genes

It is possible that the panorama views reported here reflected only a part of the reality. This is consistent with the principle of iceberg phenomenon in epidemiology, i.e. the part the reality of diseases or infections discovered by epidemiological analysis is something like the iceberg on the surface, and the majority of the iceberg is underwater and unseen. Firstly, few isolates have been reported from South America and Africa presumably due to inadequate surveillance, and influenza viruses in marine mammals also remain largely unknown. Secondly, only a few genes of many influenza viruses detected in laboratories were sequenced and reported. For example, some parts of the genomes of more than two fifths of the viral isolates, whose sequences were selected as representatives in this report, were not sequenced and reported to GenBank. As a result, we could not select the representative sequences for the six internal genes all from the same viruses. Moreover, some sequences or viral designations were reported to GenBank with errors which could distort the phylogenetic trees described here [52]. For example, the designations of two isolates within the second genotype of swine influenza viruses (i.e. the lineage S1.2.6 of the PB2 gene), A/swine/Virginia/670/1987(H1N1) and A/swine/Virginia/671/1987(H1N1), were changed in GenBank as A/swine/Italy/670/1987(H1N1) and A/swine/Italy/671/1987(H1N1), respectively, in May, 2009. The designations either before or after the changing were wrong, and this error determines whether the Eurasian lineage of swine influenza viruses has ever existed in North America or not.

In general, three types of biases could exist in the panorama trees reported here. The first is sampling bias, i.e. not all samples of type A influenza viruses circulating in the world have equal opportunities to be collected for detection. The second is detection bias, i.e. not all type A influenza viruses detected in laboratories have equal opportunities to be detected correctly and thoroughly. The third is reporting bias, i.e. not all the sequences of influenza viruses detected in laboratories have equal opportunities to be correctly reported to GenBank. It should be careful in epidemiological inference due to these biases. For example, we could not conclude that there were few influenza virus circulations in Africa or South America in the past decades because few influenza virus sequences reported there.

### The tentative universal nomenclature system of the lineages and sublineages

Classification and designation of the lineages and sublineages within type A influenza virus are essential for the studies of the viral evolution, ecology and epidemiology [1-47]. In this report, decades of lineages and sublineages within type A influenza viruses were identified, and most of them were supported by the topology of the phylogenetic trees and high bootstrap values (>70) at relevant nodes. They were also of more or less specific distribution in hosts, regions and time.

Because of the heterogeneities of the phylogenetic diversity among the six internal genes, the classification of the lineages and sublineages of the six internal genes could not be unified into the same profile.

In this report, we tentatively proposed a numerical nomenclature for the lineages and sublineages based on the six internal genes. It is informative and specific as it begins with the segment number. Because only two hierarchies are involved in the nomenclature for simplicity, some sublineages were probably evolved from other sublineages. Meanwhile, some sublineages could be further divided into some clades to describe the diversity in more details.

The nomenclature reported herein covers nearly all known type A influenza viruses, and it is easy to be expanded to meet the future evolution of the viruses. For example, a new sublineage, S1.2.7, could be assigned to the special isolate A/duck/Victoria/5384/2002(H4N8)-like viruses in Figure 1, if needed.

The proposed nomenclature is easy to remember as it is of certain orders as given in Methods. This nomenclature, if widely accepted, should facilitate international communication on the evolution, ecology and epidemiology of type A influenza viruses through the unification of current miscellaneous nomenclatures which are not only ambiguous and misleading in some cases, but also covered only a part of the diversity of type A influenza viruses. For example, the so-called "North America lineage" swine influenza viruses actually circulated widely in North America and Asia in recent years.

### Proposal to establish an ad hoc international expert group on nomenclature of influenza viruses

Influenza viruses are of high significance in human and animal health, and they are very complicated and continuously evolving. Therefore, it is desired to establish an ad hoc international expert group responsible to double check some special isolates, select the representative sequences, calculate the panorama phylogenetic trees, classify the lineages and sublineages and propose a universal nomenclature for influenza viruses and update them in time in the future. This is not that difficult as there have been many human or animal influenza reference laboratories (international and national). We recognize that our results are of reference value to the issues but also might be of some flaws.

## Conclusion

This study describes the first panorama analysis of the phylogenetic diversity and distribution of type A influenza viruses based on their six internal genes. It also proposes a tentative universal nomenclature system for the lineages and sublineages within the viral type. It provide a novel phylogenetic view (i.e. the whole view) to recognize the viruses including the origin of the pandemic A(H1N1) influenza viruses. It also presents a candidate framework to generalize the history and explore the future of the viruses.

## Methods

### Primary analysis online and selection of representative sequences

Till May 20, 2009, 7500-8800 sequences of each of the PB2, PB1, PA, NP, MP and NS genes of type A influenza virus isolates were available in GenBank. They were analyzed online using the web servers of Influenza Virus Resource in NCBI in sequence search, alignment and phylogenetic analysis [53,54]. For each gene, the sequences were divided into multiple groups according to the virus isolation time, and each group covered three years. If some time-continuous groups totally contained less than 400 sequences, they were combined into one group. If a group contained more than 600 sequences, they were further divided into several groups according to their isolation places or hosts until each group covered no more than 400 sequences. The phylogenetic relationships within each group were then analyzed using the online web servers. Lineages and sublineages of each group were classified online thereafter according to the principle stated below.

At least one representative from each of the lineages and sublineages was randomly selected with consideration of the distribution of the lineages and sublineages in hosts, isolation places and subtypes. That is to say, at least one representative was selected from those within the same sublineage provided they were distinct from the majority of the sublineage in the distribution of hosts, places, time or subtypes, and also at least one representative was selected from the majority. Most lineages or sublineages of avian influenza viruses are of multiple HA or NA subtypes, and so their representatives were selected with little consideration of subtype. On the other hand, most lineages or sublineages of human, swine or equine influenza viruses are of limited HA or NA subtypes, and thus at least one representative was selected from each of the subtypes, if available. Human and equine influenza viruses are largely of little geographical difference, and so their representatives were selected with little consideration of isolation places. However, avian influenza viruses isolated in the Eastern Hemisphere (Asia, Europe, Africa and the Pacific) were usually different from their counterparts isolated in the Western Hemisphere (North and South America), and thus at least one representative was selected from each of the hemispheres, if available. In addition, at least one representative was selected from those of similar distribution within the same sublineage provided that they were distant from others (genetic distances approximately more than 3.0%. The value is not a strict standard for such an analysis. If the value is higher, fewer representative sequences will be selected, which is of little influence on the final trees [25]). Representatives of each of the genes were favorably selected from the same viruses in order to facilitate the comparison of the phylogenetic diversity of the genes [30].

### Calculation of genetic distances

Genetic distances among the representative sequences were calculated using the model of nucleotide maximum composite likelihood including transitions and transversions using the software MEGA 4.1 [55]. The substitution rates were set equal among sites but different among lineages. Gaps were treated by pairwise-deletion, i.e. ignoring only the gaps that are involved in the comparison of a pair of sequences.

### Phylogenetic analysis

Representative sequences were aligned using the software of Clustal X [56], and the results were manually checked by eyes. Their phylogenetic relationship was analyzed using the software MEGA 4.1 with neighbor-joining method and the same parameters for the genetic distance calculation [55]. Bootstrap values were calculated out of 1000 replicates.

### Evaluation of the phylogenetic analysis results

All the sequences of each of the six internal genes of the viruses isolated from a certain host or in a certain period available in GenBank were sought as the test sequences. Their phylogenetic diversity was analyzed online using the web servers of Influenza Virus Resource in NCBI [53,54], and compared with the panorama phylogenetic trees calculated above to check whether the phylogenetic trees covered the diversity of the test sequences or not.

The phylogenetic relationships among the representatives were also calculated using RAxML version 7.0.4 with General Time Reversible (GTR) substitution matrix according to the maximum likelihood (ML) model [57,58], to evaluate the results obtained with neighbor-joining method.

### Classification of lineages and sublineages

Lineages and sublineages were classified mainly based on the topology of the phylogenetic trees, i.e. each lineage and sublineage should be of a relatively separate cluster in the trees. Distributions of the viruses in hosts, regions and time were also considered in the lineage and sublineage classification. For example, a branch within the trees might be classified as a sublineage provided it is of a distinct host even if it was of a low bootstrap value.

Designation of the lineages began with the segment number which was followed by a point and another number (e.g. S2.1 represents the first lineage of the second segment corresponding to PB1 gene), and designation of the sublineages began with the lineage designations followed by a point and a number (e.g. S2.1.1 represents the first sublineage within the lineage S2.1). The numbers within the designations of the lineages and sublineages were favorably in the order from the major to minor ones in terms of the numbers of representative sequences, and in the order of avian ones followed by human, swine, equine ones in terms of hosts, and in the order of the Western Hemisphere followed by the Eastern Hemisphere in terms of isolation places, and in the order from the past to nowadays in terms of isolation time.

## Competing interests

The authors declare that they have no competing interests.

## Authors' contributions

CJM was responsible for design and coordination of the study as well as writing the manuscript. CWC was responsible for analyzing the data and revising the manuscript. All others were responsible for sequence analysis. All authors read and approved the final manuscript.

## Supplementary Material

Additional file 1 **The designations and alignment of the representative sequences of PB2 gene**. The data could be read out with NotePad and the Mega software, and gaps are showed with hyphens. Click here for file

Additional file 2 **The designations and alignment of the representative sequences of PB1 gene**. The data could be read out with NotePad and the Mega software, and gaps are showed with hyphens. Click here for file

Additional file 3 **The designations and alignment of the representative sequences of PA gene**. The data could be read out with NotePad and the Mega software, and gaps are showed with hyphens. Click here for file

Additional file 4 **The designations and alignment of the representative sequences of NP gene**. The data could be read out with NotePad and the Mega software, and gaps are showed with hyphens. Click here for file

Additional file 5 **The designations and alignment of the representative sequences of MP gene**. The data could be read out with NotePad and the Mega software, and gaps are showed with hyphens. Click here for file

Additional file 6 **The designations and alignment of the representative sequences of NS gene**. The data could be read out with NotePad and the Mega software, and gaps are showed with hyphens. Click here for file

Additional file 7 **The original tree with virus designations of PB2 gene of type A influenza viruses**. The figure is corresponding to Figure 1. Some clades are marked with color selected at random. The viruses with exceptional distribution in hosts are marked with asterisks. Click here for file

Additional file 8 **The original tree with virus designations of PB1 gene of type A influenza viruses**. The figure is corresponding to Figure 2. Some clades are marked with color selected at random. The viruses with exceptional distribution in hosts are marked with asterisks, and two South American isolates are marked with black triangles. Click here for file

Additional file 9 **The original tree with virus designations of PA gene of type A influenza viruses**. The figure is corresponding to Figure 3. Some clades are marked with color selected at random. The viruses with exceptional distribution in hosts are marked with asterisks. Click here for file

Additional file 10 **The original tree with virus designations of NP gene of type A influenza viruses**. The figure is corresponding to Figure 4. Some clades are marked with color selected at random. The viruses with exceptional distribution in hosts are marked with asterisks. Click here for file

Additional file 11 **The original tree with virus designations of MP gene of type A influenza viruses**. The figure is corresponding to Figure 5. Some clades are marked with color selected at random. The viruses with exceptional distribution in hosts are marked with asterisks. Click here for file

Additional file 12 **The original tree with virus designations of NS gene of type A influenza viruses**. The figure is corresponding to Figure 6. Some clades are marked with color selected at random. The viruses with exceptional distribution in hosts are marked with asterisks. Click here for file

Additional file 13 **The panorama tree of HA gene of subtype H1 influenza viruses**. The tree was calculated using the methods reported previously [3]. Some clades within the tree are marked with color selected at random. Click here for file

Additional file 14 **The panorama tree of NA gene of subtype N1 influenza viruses**. The tree was calculated using the methods reported previously [3]. Some clades within the tree are marked with color selected at random. Click here for file

Additional file 15 **The panorama tree of PB2 gene of type A influenza viruses calculated using the maximum likelihood model**. The figure is corresponding to additional file 7 using the same sequence dataset. Click here for file

## References

[B1] Webster RG, Bean WJ, Gorman OT, Chambers TM, Kawaoka Y (1992). Evolution and ecology of type A influenza viruses. Microbiol Rev.

[B2] Fouchier RA, Munster V, Wallensten A, Bestebroer TM, Herfst S, Smith D, Rimmelzwaan GF, Olsen B, Osterhaus AD (2005). Characterization of a novel type A influenza virus hemagglutinin subtype (H16) obtained from black-headed gulls. J Virol.

[B3] Liu S, Ji K, Chen J, Tai D, Jiang W, Hou G, Chen J, Li J, Huang B (2009). Panorama phylogenetic diversity and distribution of type A influenza virus. PLoS ONE.

[B4] Gorman OT, Donis RO, Kawaoka Y, Webster RG (1990). Evolution of influenza A virus PB2 genes: implications for evolution of the ribonucleoprotein complex and origin of human influenza A virus. J Virol.

[B5] Ito T, Gorman OT, Kawaoka Y, Bean WJ, Webster RG (1991). Evolutionary analysis of the influenza A virus M gene with comparison of the M1 and M2 proteins. J Virol.

[B6] Gorman OT, Bean WJ, Kawaoka Y, Webster RG (1990). Evolution of the nucleoprotein gene of influenza A virus. J Virol.

[B7] Gorman OT, Bean WJ, Kawaoka Y, Donatelli I, Guo YJ, Webster RG (1991). Evolution of influenza A virus nucleoprotein genes: implications for the origins of H1N1 human and classical swine viruses. J Virol.

[B8] Zohari S, Gyarmati P, Ejdersund A, Berglöf U, Thorén P, Ehrenberg M, Czifra G, Belák S, Waldenström J, Olsen B, Berg M (2008). Phylogenetic analysis of the non-structural (NS) gene of influenza A viruses isolated from mallards in Northern Europe in 2005. Virol J.

[B9] Ludwig S, Schultz U, Mandler J, Fitch WM, Scholtissek C (1991). Phylogenetic relationship of the nonstructural (NS) genes of influenza A viruses. Virology.

[B10] Qi X, Pang B, Lu CP Genetic characterization of H1N1 swine influenza A viruses isolated in eastern China. Virus Genes.

[B11] Obenauer JC, Denson J, Mehta PK, Su X, Mukatira S, Finkelstein DB, Xu X, Wang J, Ma J, Fan Y, Rakestraw KM, Webster RG, Hoffmann E, Krauss S, Zheng J, Zhang Z, Naeve CW (2006). Large-scale sequence analysis of avian influenza isolates. Science.

[B12] Campitelli L, Donatelli I, Foni E, Castrucci MR, Fabiani C, Kawaoka Y, Krauss S, Webster RG (1997). Continued evolution of H1N1 and H3N2 influenza viruses in pigs in Italy. Virology.

[B13] Marozin S, Gregory V, Cameron K, Bennett M, Valette M, Aymard M, Foni E, Barigazzi G, Lin Y, Hay A (2002). Antigenic and genetic diversity among swine type A influenza H1N1 and H1N2 viruses in Europe. J Gen Virol.

[B14] Kuntz-Simon G, Madec F (2009). Genetic and antigenic evolution of swine influenza viruses in Europe and evaluation of their zoonotic potential. Zoonoses Public Health.

[B15] Guan Y, Shortridge KF, Krauss S, Li PH, Kawaoka Y, Webster RG (1996). Emergence of avian H1N1 influenza viruses in pigs in China. J Virol.

[B16] Chambers TM, Hinshaw VS, Kawaoka Y, Easterday BC, Webster RG (1991). Influenza viral infection of swine in the United States 1988-1989. Arch Virol.

[B17] Brown IH, Hill ML, Harris PA, Alexander DJ, McCauley JW (1997). Genetic characterisation of a type A influenza virus of unusual subtype (H1N7) isolated from pigs in England. Arch Virol.

[B18] Bryant NA, Rash AS, Russell CA, Ross J, Cooke A, Bowman S, MacRae S, Lewis NS, Paillot R, Zanoni R, Meier H, Griffiths LA, Daly JM, Tiwari A, Chambers TM, Newton JR, Elton DM (2009). Antigenic and genetic variations in European and North American equine influenza virus strains (H3N8) isolated from 2006 to 2007. Vet Microbiol.

[B19] Schafer JR, Kawaoka Y, Bean WJ, Suss J, Senne D, Webster RG (1993). Origin of the pandemic 1957 H2 type A influenza virus and the persistence of its possible progenitors in the avian reservoir. Virology.

[B20] Wahlgren J, Waldenström J, Sahlin S, Haemig PD, Fouchier RA, Osterhaus AD, Pinhassi J, Bonnedahl J, Pisareva M, Grudinin M, Kiselev O, Hernandez J, Falk KI, Lundkvist A, Olsen B (2008). Gene segment reassortment between American and Asian lineages of avian influenza virus from waterfowl in the Beringia Area. Vector Borne Zoonotic Dis.

[B21] Xu KM, Li KS, Smith GJ, Li JW, Tai H, Zhang JX, Webster RG, Peiris JS, Chen H, Guan Y (2007). Evolution and molecular epidemiology of H9N2 type A influenza viruses from quail in southern China, 2000 to 2005. J Virol.

[B22] Kawaoka Y, Krauss S, Webster RG (1989). Avian-to-human transmission of the PB1 gene of influenza A viruses in the 1957 and 1968 pandemics. J Virol.

[B23] Widjaja L, Krauss SL, Webby RJ, Xie T, Webster RG (2004). Matrix gene of type A influenza viruses isolated from wild aquatic birds: ecology and emergence of type A influenza viruses. J Virol.

[B24] Zell R, Motzke S, Krumbholz A, Wutzler P, Herwig V, Dürrwald R (2008). Novel reassortant of swine influenza H1N2 virus in Germany. J Gen Virol.

[B25] Bush RM, Smith CB, Cox NJ, Fitch WM (2000). Effects of passage history and sampling bias on phylogenetic reconstruction of human influenza A evolution. Proc Natl Acad Sci USA.

[B26] Fitch WM, Bush RM, Bender CA, Cox NJ (1997). Long term trends in the evolution of H(3) HA1 human influenza type A. Proc Natl Acad Sci USA.

[B27] Guo Y, Wang M, Kawaoka Y, Gorman O, Ito T, Saito T, Webster RG (1992). Characterization of a new avian-like type A influenza virus from horses in China. Virology.

[B28] Bahl J, Vijaykrishna D, Holmes EC, Smith GJ, Guan Y (2009). Gene flow and competitive exclusion of avian influenza A virus in natural reservoir hosts. Virology.

[B29] Macken CA, Webby RJ, Bruno WJ (2006). Genotype turnover by reassortment of replication complex genes from avian type A influenza virus. J Gen Virol.

[B30] Bragstad K, Nielsen LP, Fomsgaard A (2008). The evolution of human influenza A viruses from 1999 to 2006: a complete genome study. Virol J.

[B31] Koehler AV, Pearce JM, Flint PL, Franson JC, Ip HS (2008). Genetic evidence of intercontinental movement of avian influenza in a migratory bird: the northern pintail (Anas acuta). Mol Ecol.

[B32] Cattoli G, Monne I, Fusaro A, Joannis TM, Lombin LH, Aly MM, Arafa AS, Sturm-Ramirez KM, Couacy-Hymann E, Awuni JA, Batawui KB, Awoume KA, Aplogan GL, Sow A, Ngangnou AC, El Nasri Hamza IM, Gamatié D, Dauphin G, Domenech JM, Capua I (2009). Highly pathogenic avian influenza virus subtype H5N1 in Africa: a comprehensive phylogenetic analysis and molecular characterization of isolates. PLoS ONE.

[B33] Kida H, Shortridge KF, Webster RG (1988). Origin of the hemagglutinin gene of H3N2 influenza viruses from pigs in China. Virology.

[B34] Shin JY, Song MS, Lee EH, Lee YM, Kim SY, Kim HK, Choi JK, Kim CJ, Webby RJ, Choi YK (2006). Isolation and characterization of novel H3N1 swine influenza viruses from pigs with respiratory diseases in Korea. J Clin Microbiol.

[B35] Chutinimitkul S, Thippamom N, Damrongwatanapokin S, Payungporn S, Thanawongnuwech R, Amonsin A, Boonsuk P, Sreta D, Bunpong N, Tantilertcharoen R, Chamnanpood P, Parchariyanon S, Theamboonlers A, Poovorawan Y (2008). Genetic characterization of H1N1, H1N2 and H3N2 swine influenza virus in Thailand. Arch Virol.

[B36] Yu H, Hua RH, Zhang Q, Liu TQ, Liu HL, Li GX, Tong GZ (2008). Genetic evolution of swine type A influenza (H3N2) viruses in China from 1970 to 2006. J Clin Microbiol.

[B37] Sun L, Zhang G, Shu Y, Chen X, Zhu Y, Yang L, Ma G, Kitamura Y, Liu W (2009). Genetic correlation between H3N2 human and swine influenza viruses. J Clin Virol.

[B38] WHO/OIE/FAO H5N1 Evolution Working Group (2009). Toward a unified nomenclature system for highly pathogenic avian influenza virus (H5N1). Emerg Infect Dis.

[B39] Muller I, Jaureguiberry B, Valenzuela PD (2005). Isolation, sequencing and phylogenetic analysis of the hemagglutinin, neuraminidase and nucleoprotein genes of the Chilean equine influenza virus subtypes H7N7 and H3N8. Biol Res.

[B40] Solovyov A, Palacios G, Briese T, Lipkin WI, Rabadan R (2009). Cluster analysis of the origins of the new influenza A(H1N1) virus. Euro Surveill.

[B41] Centers for Disease Control (2009). Swine influenza A (H1N1) infection in two children-Southern California, March-April 2009. MMWR.

[B42] Novel Swine-Origin Influenza A (H1N1) Virus Investigation Team (2009). Emergence of a novel swine-origin influenza A (H1N1) virus in humans. N Engl J Med.

[B43] Smith GJ, Vijaykrishna D, Bahl J, Lycett SJ, Worobey M (2009). Origins and evolutionary genomics of the 2009 swine-origin H1N1 influenza A epidemic. Nature.

[B44] Trifonov V, Khiabanian H, Rabadan R (2009). Geographic dependence, surveillance, and origins of the 2009 influenza A (H1N1) virus. N Engl J Med.

[B45] Chen J, Sun Y, Liu S, Jiang W, Chen J, Hou G, Li J (2009). Origin and future distribution of the new A(H1N1) influenza virus emerging in North America in 2009. Chinese Sci Bull.

[B46] Garten RJ, Davis CT, Russell CA, Shu B, Lindstrom S, Balish A, Sessions WM, Xu X, Skepner E, Deyde V, Okomo-Adhiambo M, Gubareva L, Barnes J, Smith CB, Emery SL, Hillman MJ, Rivailler P, Smagala J, de Graaf M, Burke DF, Fouchier RA, Pappas C, Alpuche-Aranda CM, López-Gatell H, Olivera H, López I, Myers CA, Faix D, Blair PJ, Yu C, Keene KM, Dotson PD, Boxrud D, Sambol AR, Abid SH, St George K, Bannerman T, Moore AL, Stringer DJ, Blevins P, Demmler-Harrison GJ, Ginsberg M, Kriner P, Waterman S, Smole S, Guevara HF, Belongia EA, Clark PA, Beatrice ST, Donis R, Katz J, Finelli L, Bridges CB, Shaw M, Jernigan DB, Uyeki TM, Smith DJ, Klimov AI, Cox NJ (2009). Antigenic and genetic characteristics of swine-origin 2009 A(H1N1) influenza viruses circulating in humans. Science.

[B47] Smith GJ, Vijaykrishna D, Bahl J, Lycett SJ, Worobey M, Pybus OG, Ma SK, Cheung CL, Raghwani J, Bhatt S, Peiris JS, Guan Y, Rambaut A (2009). Origins and evolutionary genomics of the 2009 swine-origin H1N1 influenza A epidemic. Nature.

[B48] Chen JM, Guo YJ, Wu KY, Guo JF, Wang M, Dong J, Zhang Y, Li Z, Shu YL (2007). Exploration of the emergence of the Victoria lineage of influenza B virus. Arch Virol.

[B49] Zhou NN, Senne DA, Landgraf JS, Swenson SL, Erickson G, Rossow K, Liu L, Yoon K, Krauss S, Webster RG (1999). Genetic reassortment of avian, swine, and human influenza A viruses in American pigs. J Virol.

[B50] Ito T, Couceiro JN, Kelm S, Baum LG, Krauss S, Castrucci MR, Donatelli I, Kida H, Paulson JC, Webster RG, Kawaoka Y (1998). Molecular basis for the generation in pigs of influenza A viruses with pandemic potential. J Virol.

[B51] Suzuki Y, Ito T, Suzuki T, Holland RE, Chambers TM, Kiso M, Ishida H, Kawaoka Y (2000). Sialic acid species as a determinant of the host range of influenza A viruses. J Virol.

[B52] Krasnitz M, Levine AJ, Rabadan R (2008). Anomalies in the influenza virus genome database: new biology or laboratory errors?. J Virol.

[B53] Bao Y, Bolotov P, Dernovoy D, Kiryutin B, Zaslavsky L, Tatusova T, Ostell J, Lipman D (2008). The influenza virus resource at the National Center for Biotechnology Information. J Virol.

[B54] Zaslavsky L, Bao Y, Tatusova TA (2008). Visualization of large influenza virus sequence datasets using adaptively aggregated trees with sampling-based subscale representation. BMC Bioinformatics.

[B55] Tamura K, Dudley J, Nei M, Kumar S (2007). MEGA4: Molecular Evolutionary Genetics Analysis (MEGA) software version 4.0. Mol Biol Evol.

[B56] Larkin MA, Blackshields G, Brown NP, Chenna R, McGettigan PA, McWilliam H, Valentin F, Wallace IM, Wilm A, Lopez R, Thompson JD, Gibson TJ, Higgins DG (2007). Clustal W and Clustal X version 2.0. Bioinformatics.

[B57] Stamatakis A (2006). RAxML-VI-HPC: maximum likelihood-based phylogenetic analyses with thousands of taxa and mixed models. Bioinformatics.

[B58] Stamatakis A, Hoover P, Rougemont J (2008). A rapid bootstrap algorithm for the RAxML Web servers. Syst Biol.

