# Supplementary figures and images for: Panorama phylogenetic diversity and distribution of type A influenza viruses based on their six internal gene sequences
Source: Virol J. 2009 Sep 8;6:137. doi: 10.1186/1743-422X-6-137 (PMC2746212; doi:10.1186/1743-422X-6-137)

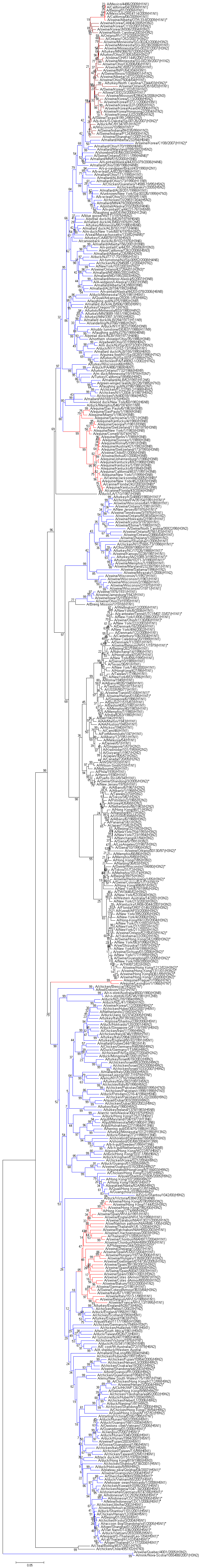

Supplement: Additional file 7 — The original tree with virus designations of PB2 gene of type A influenza viruses. The figure is corresponding to Figure 1. Some clades are marked with color selected at random. The viruses with exceptional distribution in hosts are marked with asterisks. [file 1743-422X-6-137-S7.tiff]

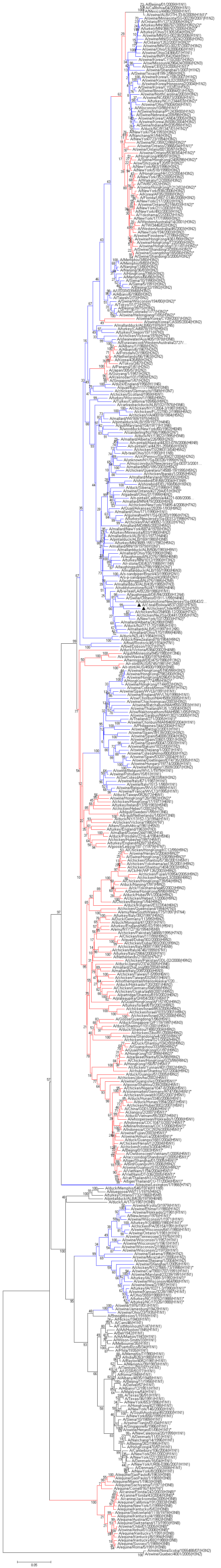

Supplement: Additional file 8 — The original tree with virus designations of PB1 gene of type A influenza viruses. The figure is corresponding to Figure 2. Some clades are marked with color selected at random. The viruses with exceptional distribution in hosts are marked with asterisks, and two South American isolates are marked with black triangles. [file 1743-422X-6-137-S8.tiff]

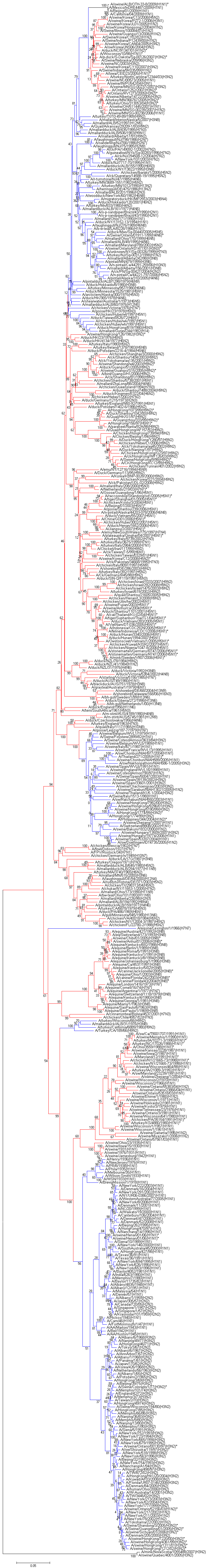

Supplement: Additional file 9 — The original tree with virus designations of PA gene of type A influenza viruses. The figure is corresponding to Figure 3. Some clades are marked with color selected at random. The viruses with exceptional distribution in hosts are marked with asterisks. [file 1743-422X-6-137-S9.tiff]

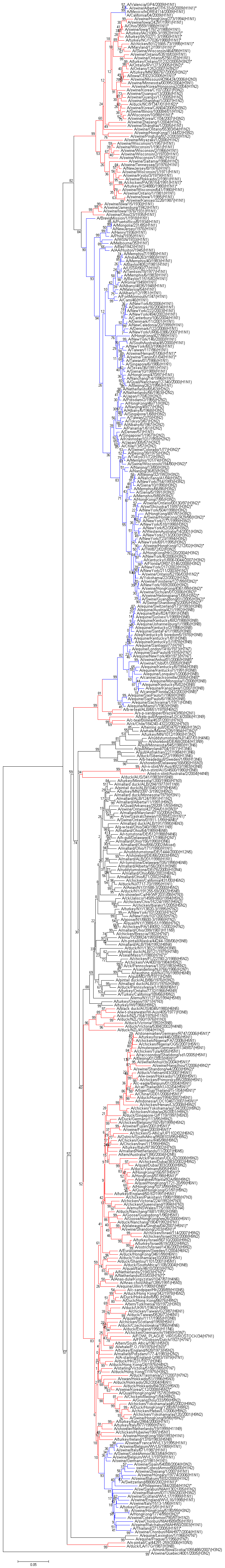

Supplement: Additional file 10 — The original tree with virus designations of NP gene of type A influenza viruses. The figure is corresponding to Figure 4. Some clades are marked with color selected at random. The viruses with exceptional distribution in hosts are marked with asterisks. [file 1743-422X-6-137-S10.tiff]

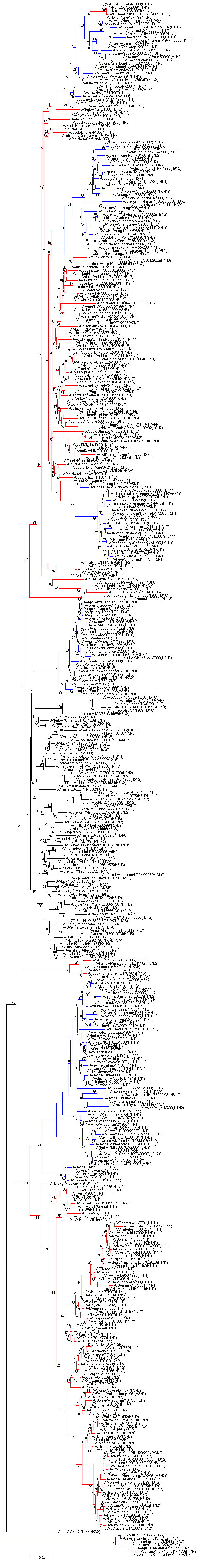

Supplement: Additional file 11 — The original tree with virus designations of MP gene of type A influenza viruses. The figure is corresponding to Figure 5. Some clades are marked with color selected at random. The viruses with exceptional distribution in hosts are marked with asterisks. [file 1743-422X-6-137-S11.tiff]

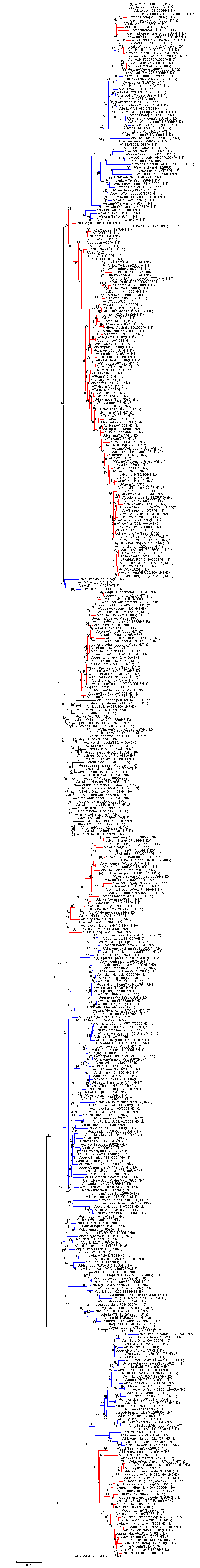

Supplement: Additional file 12 — The original tree with virus designations of NS gene of type A influenza viruses. The figure is corresponding to Figure 6. Some clades are marked with color selected at random. The viruses with exceptional distribution in hosts are marked with asterisks. [file 1743-422X-6-137-S12.tiff]

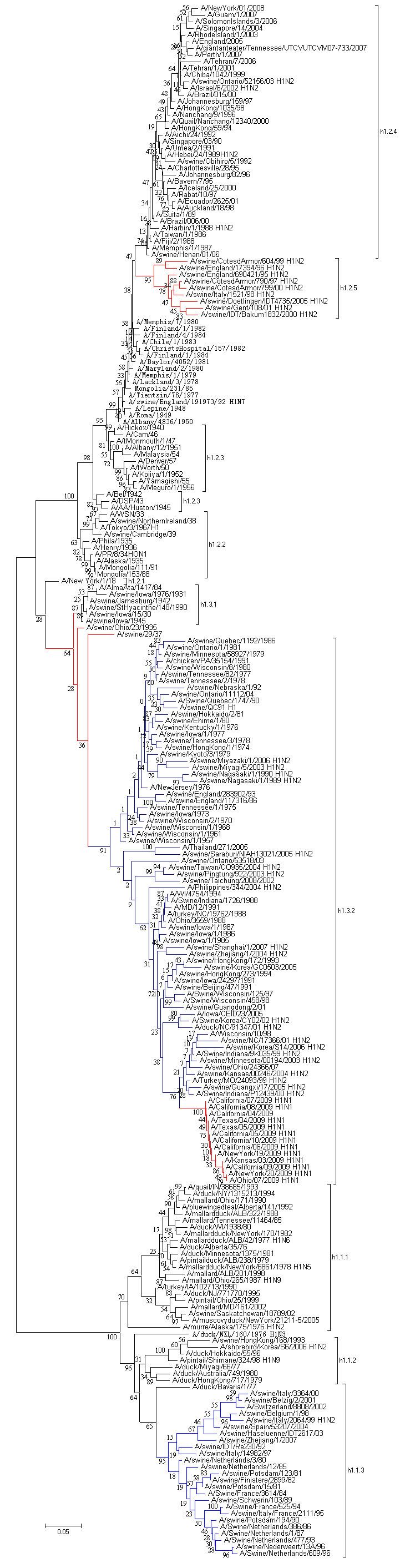

Supplement: Additional file 13 — The panorama tree of HA gene of subtype H1 influenza viruses. The tree was calculated using the methods reported previously [3]. Some clades within the tree are marked with color selected at random. [file 1743-422X-6-137-S13.tiff]

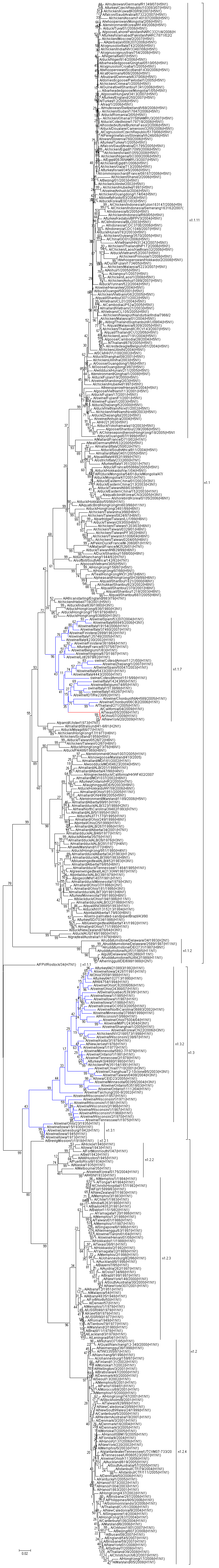

Supplement: Additional file 14 — The panorama tree of NA gene of subtype N1 influenza viruses. The tree was calculated using the methods reported previously [3]. Some clades within the tree are marked with color selected at random. [file 1743-422X-6-137-S14.tiff]

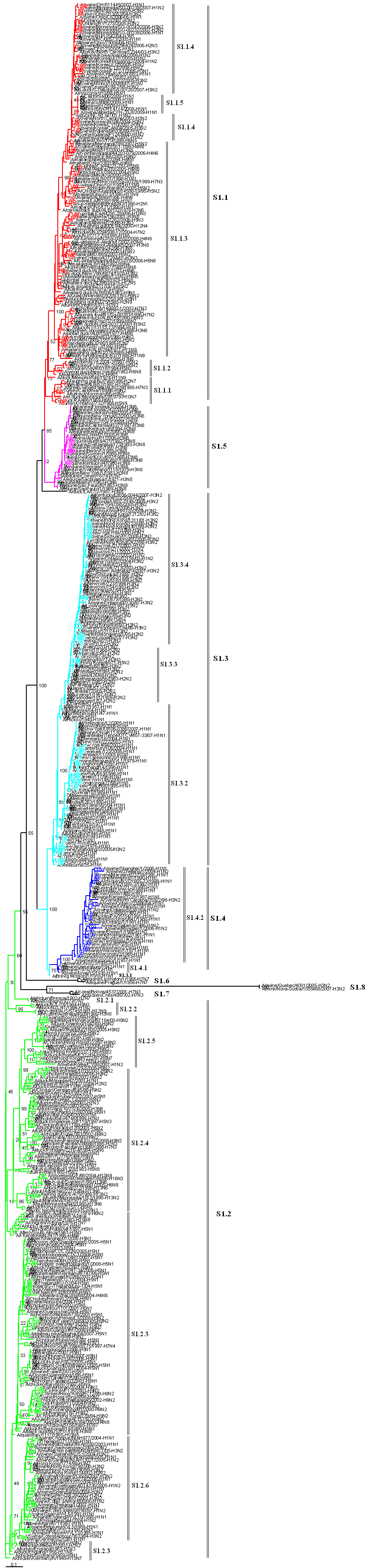

Supplement: Additional file 15 — The panorama tree of PB2 gene of type A influenza viruses calculated using the maximum likelihood model. The figure is corresponding to additional file 7 using the same sequence dataset. [file 1743-422X-6-137-S15.tiff]
